# Adoption of McKinsey 7s Model in Development of Strategies to Enhance the Utilisation of Digital Health in Early Detection and Treatment of Pre‐Eclampsia by Gravid Women at the Rural Context: Mixed‐Method Study

**DOI:** 10.1002/nop2.70162

**Published:** 2025-03-08

**Authors:** Mxolisi Welcome Ngwenya, Livhuwani Muthelo, Tebogo Maria Mothiba

**Affiliations:** ^1^ Department of Nursing Science University of Limpopo Polokwane Limpopo South Africa; ^2^ Faculty of Health Science University of Limpopo Polokwane Limpopo South Africa

**Keywords:** digital health, early detection, gravid and pre‐eclampsia, treatment

## Abstract

**Aim:**

To develop evidence‐based strategies to enhance digital health use in the early detection and treatment of pre‐eclampsia by pregnant women in primary healthcare facilities in a South African context.

**Design:**

The mixed convergent parallel design was used for the main study. The quantitative and qualitative data analysed served as the basis for this study.

**Methods:**

The McKinsey 7s model was used to guide the development of evidence‐based strategies to enhance the utilisation of digital health in early detection and treatment of pre‐eclampsia by gravid women within a mixed convergent parallel research design.

**Results:**

Six evidence‐based strategies were developed. The strategies focused on improving knowledge, training and professional development, awareness and validation of digital health.

**Public Contribution:**

Although the study focused in the primary healthcare facilities in Mpumalanga province, the evidence‐based strategies developed promote the breaking of the status quo in current practice. Integration of traditional clinical practice and digital health practice could possibly improve the quality of care received by gravid women and most likely, improve the knowledge of the gravid women with regard to their conditions. Furthermore, their implementation could possibly improve the proper practicing and implementation of digital health in clinical practice.

## 1 Introduction

Digital health is a growing branch in the health field. The significant use of digital health for the prevention and treatment of chronic and acute conditions has been reported to be effective. The explosive growth of digital health use worldwide has shown that change is inevitable. Digital health changed care models and changed the focus in health systems towards patient‐centred healthcare, particularly in limited resource settings (Mitchell and Kan 2019). The study by Aranda‐Jan et al. (2014) substantiated the use of digital health initiatives such as mobile health technology (mHealth) has also been shown to be rapidly expanding in developing countries in Africa. Furthermore, mHealth in Africa is considered an innovative approach in providing healthcare services and has proven to be the fast‐growing technological field with research opportunities that include assessing the scaling up of mHealth projects, cost‐effectiveness and its impacts on the health system overall.

A narrative review in low‐income countries conducted by Osei and Mashamba‐Thompson (2021) corroborated those advances in digital health such as mobile technology and other applications is driving the transformation in the delivery of health systems globally. Integration of digital health into the delivery of current clinical services provides new channels in quality care of the delivery of health services (Adepoju et al. 2017). The introduction of digital health in maternal services is also regarded as a strategic plan to minimise maternal mortalities associated with pregnancy complications such as pre‐eclampsia. The use of digital health during pregnancy has been associated with a reduction in hospitalisation as a result of pregnancy‐related complications. Despite the digital health initiatives in maternal health services, a significant number of studies identified several impedimental factors for the implementation of the initiatives of both users; pregnant women and healthcare providers. The impediments included poor coordination and communications, unreliable technology, lack of knowledge of technology, people's financial status and untrained personnel (Kiberu et al. 2017; Littman‐Quinn et al. 2013; Ohia et al. 2023). As it may be, strategies and guidelines for the implementation of digital health at all levels of health must be in place to improve the effectiveness and efficient use of digital health by pregnant women and healthcare providers.

However, the general studies recognised pertinent to digital health in maternal services outlined the importance of digital health in the effectiveness and efficiency of maternal healthcare services. These included reduced admission among pre‐eclampsia women, increased awareness of pregnancy‐related complications, lifestyle modification, symptom control and improved accessibility (Keasberry et al. 2017; Mackintosh et al. 2021; West 2015).

In South Africa, hypertension is one of the common pregnancy complications that result in maternal and perinatal morbidity and mortality. Accounting for approximately 661 as indicated in the saving mothers report triennium report 2014–2016 (saving mothers, 2019). This shows that despite the action of the last triennium, maternal mortality increased by 21 from the last triennium. One of the leading primary causes is pre‐eclampsia. This was supported by a study conducted in Limpopo province of 14,685 live births, 232 women died and the primary causes were pre‐eclampsia and obstetric haemorrhage (Ntuli et al. 2019). However, it is clear from evidence in the literature that South Africa had limited studies on the utilisation of digital health initiatives in the early detection and treatment of pre‐eclampsia. These clearly show the significant need to increase awareness of digital health in mass communities and pregnant women, as this could possibly lead to the achievement of the sustainable development goals (SDG) 3.1 target, which is reduction of maternal and infant mortality (Ghulmiyyah and Sibai 2012).

The current article is part of large research project aimed to develop evidence‐based strategies to enhance the utilisation of digital health in early detection and treatment of pre‐eclampsia by gravid women in Emalahleni local municipality of Mpumalanga province. A mixed research methodology was used to gather the perspectives of midwives and gravid women who had pre‐eclampsia looking at their knowledge and barriers to digital health use in early detection and treatment of pre‐eclampsia. The analysed data lead to the development of evidence‐based strategies to enhance the utilisation of digital health in early detection and treatment of pre‐eclampsia by gravid women.

### 1.1 McKinsey 7s Model

A McKinsey 7s model was used to guide the study in developing evidence‐based strategies to enhance the use of digital health in early detection and treatment of pre‐eclampsia in PHC facilities. A McKinsey 7s model is an instrument that examines and analyses an organisation design taking into consideration the seven key components namely; structure, strategy, systems, style, staff, skills and shared values. Furthermore, one of McKinsey 7s model uses is to assess and identify areas that may require change in a future (Nejad et al. 2015; Jurevicius 2013). Primary healthcare (PHC) facilities were made the cornerstone of achieving the SDGs by 2030 by the global health community. One of the SDGs is to ensure healthy lives and promote health for all ages; this includes reducing maternal and perinatal mortality (Asi and Williams 2018; Statssa 2019). In the context of the main study, a convergent parallel research design was implemented to identify the knowledge gaps and barriers among midwives and gravid women in the use of digital health in early detection and treatment of pre‐eclampsia in primary care (PHC) facilities. This allowed the authors to identify areas that require changes within the facilities and implement the change through the development of strategies (Nejad et al. 2015).

**FIGURE 1:**
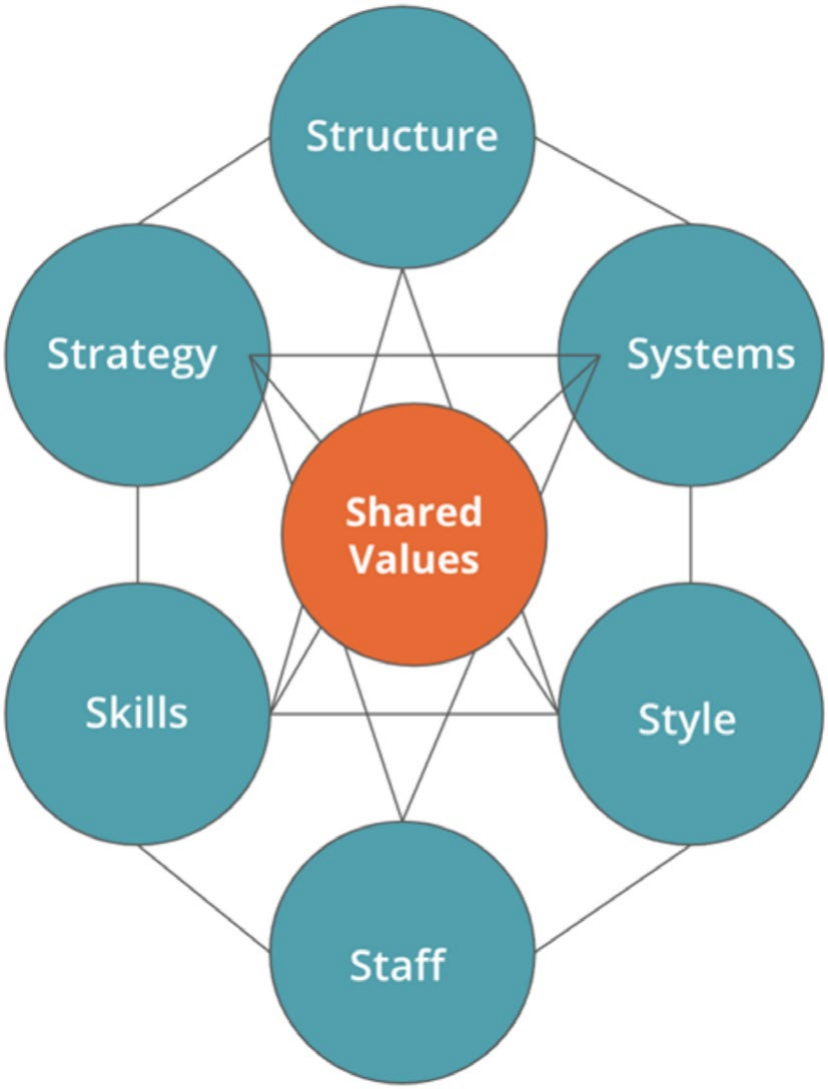
Schematic diagram of the interaction of the McKinsey 7s model (Sammut‐Bonnici and McGee 2014).

The McKinsey 7s model components are interdependent and one of the steps when applying the model is to identify areas that are not appropriately aligned by looking at the 7s components if they are aligned to each other (Nejad et al. 2015; Sammut‐Bonnici and McGee 2014). In the context of the study, three components of the model were not aligned. Inconsistencies, gaps and weaknesses were identified in the systems, staff, skills and style of the PHC facilities. The article focused on the components that aligned with the study and had gaps. Figure 1 displays a schematic diagram of the McKinsey 7s models and interactions of its components as suggested by Sammut‐Bonnici and McGee (2014).

## 2 Methods

The larger study investigated the knowledge and barriers to digital health utilisation in the early detection and treatment of pre‐eclampsia by midwives and gravid women. Thus, to develop evidence‐based strategies to improve the use of digital health in the early detection and treatment of pre‐eclampsia by gravid women. It sampled the population of midwives and gravid women living with pre‐eclampsia. In the quantitative strand, to guarantee equal participation possibilities, the authors selected midwives using the basic random sample probability method. The study sampling plan ensured that each participant had an equal and independent opportunity to participate in the investigation. The study sample size, calculated using the Slovin formula, was 109. However, because some people did not participate in the study and because some questionnaires were incomplete. The 102 respondents who completed the questionnaires made up the sample size. Self‐developed questionnaires were used for data collection. The questionnaires were developed based on existing literature and highlighted the positive implications of digital health, as well as the barriers and challenges of digital health. After consulting with professors and experts in the intended field of study, the questionnaires were examined and corrected for errors and discrepancies before using the instrumentation in data collection. The reliability of the questionnaire was then tested using Cronbach alpha. The overall Cronbach alpha of the standardised items was 0.624. This indicates that the instrument tool is moderately reliable and acceptable, as stated by Daud et al. (2018). A descriptive statistical analysis was used for the analysis of the quantitative data.

In the qualitative strand, gravid women living with pre‐eclampsia were purposively selected. Semi‐structured interviews were adopted as a data collection tool. Data saturation was reached with the 10th gravid women. Open‐tesch coding was used for data analysis. Since the study adopted a mixed convergent parallel mixed method, data was collected simultaneously from midwives and gravid women over a period of 2–3 months in 2022. The data were then merged and analysed using a mixed‐method cross case comparison analysis strategy.

## 3 Results

The results of the study are presented and interpreted below. First, a brief summary of the qualitative and quantitative results is presented. Then, the integration of the results with the Mckinsey 7s model is discussed.

### 3.1 Quantitative Strand

Quantitative results are presented in two sections, namely, knowledge and importance of digital health and barriers to digital health. Table 1 represents the knowledge and importance of digital health and Table 2 presents the barriers to digital health utilisation for early detection and management of pre‐eclampsia.

**TABLE 1:** The knowledge and importance of digital health findings (Ngwenya, 2023).

Knowledge and importance	Disagree		Agree		Total
	*F*	%	*F*	%	*N* (%)
Digital health is the use of digital, mobile and wireless technologies for health	18	17.6	84	82.4	102 (100)
2 The purpose of digital health is to minimise maternal mortality and morbidity through active communication with pregnant women using mobile initiatives such as Mom Connect	14	13.7	88	86.3	102 (100)
3 Digital health apps to assist midwives in the accurate diagnosis of patients with pre‐eclampsia	17	16.7	85	83.3	102 (100)
4 Digital health helps me make an informed decision on when to refer the patient to a higher facility	21	20.6	81	79.4	102 (100)
5 The use of digital health helps detect early conditions such as pre‐eclampsia in pregnant women	18	17.6	84	82.4	102 (100)
6 In the pandemic era, digital health helps to curve the spread of COVID‐19	13	12.7	89	87.3	102 (100)
7 Digital health provides midwives with up‐to‐date treatment for pre‐eclampsia	18	17.6	84	82.4	102 (100)
8 Digital health apps provide nurses with nutritional advice for women with pre‐eclampsia	16	15.7	86	84.3	102 (100)
9 Digital health provides midwives with a proper assessment guide for pre‐eclampsia women	17	16.7	85	83.3	102 (100)
10 Can you register a multiparity to mom connect each pregnancy?	13	12.7	89	87.3	102 (100)
11 Mom Connect has no effect on pre‐eclampsia women	37	36.3	65	63.7	102 (100)
12 I advise the patient about the use of digital health apps	17	16.7	85	83.3	102 (100)
13 Digital health initiatives such as mom connect inform pre‐eclampsia patients on how to care for themselves and the fetus	8	7.8	94	92.2	102 (100)
14 Digital health apps help identify women at risk of pre‐eclampsia early	28	27.5	74	72.5	102 (100)
15 I advise pre‐eclampsia women to consult the Internet for information about pre‐eclampsia danger signs	46	45.1	56	54.9	102 (100)

**TABLE 2:** Shows barriers to the use of digital health (Ngwenya, 2023).

	Disagree		Agree		Total
	*F*	%	*F*	%	*N* (%)
Barriers to digital health use					
16 Lack of technology validation	26	25.5	76	74.5	102 (100)
17 Patients do not have a mobile phone.	63	61.8	39	38.2	102 (100)
18 Poor network connection in the facility	38	37.3	64	62.7	102 (100)
19 Patients not interested in mHealth initiatives	70	68.6	32	31.4	102 (100)
20 Interferes with the patient–nurse relationship	54	52.9	48	47.1	102 (100)
21 I find digital health to be unreliable.	67	65.7	35	34.3	102 (100)
22 Most of the patients are technologically illiterate.	64	62.7	38	37.3	102 (100)
23 Lack of awareness of digital health	26	25.5	76	74.5	102 (100)
24 You don't have enough time to use digital health during patient consultation.	30	29.4	72	70.6	102 (100)
25 I find the digital health app difficult to use.	59	57.8	43	42.2	102 (100)
26 Digital health does not cover relevant treatment for pre‐eclampsia.	74	72.5	28	27.5	102 (100)
27 I don't have a smartphone to access digital health apps	78	76.5	24	23.5	102 (100)
28 The facility does not have electronic devices such as tablets, Wi‐Fi, or computers.	39	38.2	63	61.8	102 (100)
29 Electronic health record system not implemented in the facility	58	56.9	44	43.1	102 (100)
30 Digital health does not have enough information; therefore, I don't use it.	69	67.6	33	32.4	102 (100)
31 Lack of policies, programmes and strategies to optimise utilisation in digital health among pregnant women within the facility	46	45.1	56	54.9	102 (100)
32 Digital health requires more mental efforts.	74	72.5	28	27.5	102 (100)
33 Experience electricity problems in the area	28	27.5	74	72.5	102 (100)
34 Overcrowding within the facility	27	26.5	75	73.5	102 (100)
Staffing and staff development					
35 Shortage of staff in the facility	28	27.5	74	72.5	102 (100)
36 Absenteeism high in the facility	66	64.7	36	35.3	102 (100)
37 Trained in digital health in the past 2 years	96	94.1	6	5.9	102 (100)
38 Trained in authorised applications for digital health	96	94.1	6	5.9	102 (100)
39 In‐service training of pre‐eclampsia	19	18.6	83	81.4	102 (100)
40 Attendance of perinatal mortality meetings	14	13.7	88	86.3	102 (100)
41 Allowed to develop a higher educational level in maternal and child services and nursing informatics.	24	23.5	78	76.5	102 (100)
42 Poor patient midwives ratio	40	39.2	62	60.8	102 (100)
43 Too much work load	22	21.6	80	78.4	102 (100)

Table 1 shows that midwives have significant knowledge of the use of digital health in the early detection and treatment of pre‐eclampsia. Most midwives (82.4%) agreed that digital health is the use of digital, mobile and wireless technologies for health. Furthermore, the study revealed that 88 (86.3%) midwives noted that the purpose of digital health is to minimise maternal mortality and morbidity through active communication with pregnant women using mobile initiatives such as Mom Connect. Furthermore, 85 (83.3%) reported that digital health apps help them in the accurate diagnosis of patients with pre‐eclampsia in their workplace. For example, 81 (79.4%) midwives also reported that the use of digital health helps them make an informed decision on whether to refer a patient to another facility for further management. Midwives (82.4%) revealed that digital health use is significant in detecting early conditions such as pre‐eclampsia among pregnant women.

The study findings in Table 2 show that midwives face multiple barriers when it comes to using digital health to treat pre‐eclampsia. For example, the study findings showed that the primary barriers to using digital health to treat pre‐eclampsia were lack of technological validation (74.5%), poor connection to the facility network (62.7%) and lack of knowledge of digital health initiatives (74.5%). In addition to these barriers, the study's results showed that 70.6% of midwives lack the time necessary to employ digital health during patient consultations. However, additional barriers, such as a lack of policies, programmes and strategies to maximise the use of digital health by pregnant women within the facility (54.9%), overcrowding (73.5%) and electrical problems, also contributed to the failure of midwives to use digital health to treat preeclampsia. Finally, most midwives (61.8%) also mentioned that there are no laptops, tablets or Wi‐Fi in the facility. Table 2 further indicates that 72.5% of the midwives agreed that the shortage of staff in the facility was also an impediment to the use of digital health. Above all, 94.1% and 94.1% of midwives, respectively, disagreed that they had received training in digital health within the previous 2 years and approved digital health applications. However, 81.4% and 86.3%, respectively, acknowledged having attended perinatal meetings and undergone in‐service training on pre‐eclampsia. Furthermore, 76.5% of midwives mentioned that they were granted permission to pursue higher education in the fields of nursing informatics and maternity and child services. Finally, most midwives agreed that the use of digital health in the treatment of pre‐eclampsia was hampered by a low patient‐to‐midwife ratio (60.8%) and excessive workload (78.4%).

### 3.2 Qualitative Strand

Table 3 briefly shows the themes and subthemes that emerged from the qualitative strand.

**TABLE 3:** Qualitative findings as shown by Ngwenya (2023).

Themes	Subthemes
The Role of Knowledge About Digital Health in its Utilisation	1.1 Digital health as a tool to enhance access to information 1.2 Low awareness of the availability of digital health services and alternative sources of information 1.3 Knowledge versus lack of knowledge about the use of digital health and alternative sources of information 1.4 Other non‐digital sources of information 1.5 The role of family and friends in supplementing the information from PHC
2 Barriers to Digital Health Use	2.1 The challenge of poor network connectivity and lack of technological knowledge 2.2 The lack of trust and validation of digital health initiatives is a concern 2.3 Poor socioeconomic status and access to digital health 2.4 Lack of support for the use of available digital health services

### 3.3 Presentation of Mixed Findings

Using a mixed‐method cross‐case comparison analytical strategy, the authors were able to identify inconsistencies and consistencies in all cases and create explanations for why they occur. Cross‐case comparison is a mixed‐method analytical technique that consolidates quantitative and qualitative data by building holistic, internally coherent profiles that are used to test or expand upon quantitatively or qualitatively derived themes for comparison purposes (Creamer 2017). This enabled the authors to identify the overlapping constructs after merging the findings. These further produced the main conclusions of the study and the main themes were formed (Creamer 2017). Table 4 presents the main themes of the study along with the qualitative quotes.

**TABLE 4:** Principal themes (Ngwenya, 2023).

Principal themes	Quantitative data	Qualitative extracts
Knowledge and importance of digital health utilisation in the management of pre‐eclampsia	Quantitative results reported 83.3% digital health apps to assist midwives help in accurate diagnosing of pre‐eclampsia Patients	The problem is that I don't have a phone that am currently using. Therefore, I don't use digital health Initiatives
Shortage of digital and human resources	Quantitative results reported that there is overcrowding in the facility (73.5%), Staff shortage (72.5%) and too much workload (78.4%)	The problem is that I don't have a phone that am currently using. Therefore, I don't use digital health initiatives
Power supply inconveniences impacting network connectivity	Quantitative results reported that midwives experience electricity (72.5%) and 62.7% reported experiencing poor network connectivity	Sometimes I'd face network connectivity problems when trying to access information about my pregnancy and pre‐eclampsia, especially during load shedding
Lack of awareness regarding the use of digital health in early detection and treatment of pre‐eclampsia	Quantitative results indicated that lack of awareness is a barrier to digital health utilisation (74.5%)	There was no apps I used instead, I googled the website pages called babies mommy and etc. You know when you go to Google and search for that specific content you want, it shows you a variety of options. Then I would choose flow and also mom & baby, calendar, I visit those a lot
Lack of training on the use of digital health	The quantitative results revealed that 94.1% of midwives received no training on digital health	I have never received any support with regards to the use of digital health initiatives. They never taught me anything about digital health initiatives that may help me with my pregnancy
Lack of validation and policies of digital health initiatives	Quantitative results reported that 74.5% of midwives indicated that lack of validation is a barrier to digital health	The thing is that for me, I was never sure which website to use and trust, because I was never taught about it at the clinic

#### 3.3.1 Theme 1: Knowledge and Importance of Digital Health Utilisation in the Management of Pre‐Eclampsia

The quantitative findings revealed that midwives have adequate knowledge of the use of digital health in early detection and treatment of pre‐eclampsia. Based on knowledge and importance variables, 82.4% of midwives indicated that digital health is the use of digital, mobile and wireless technologies for health. Most midwives (86.3%) reported that the purpose of digital health is to minimise maternal mortality and morbidity through active communication with women with serious medical conditions using mobile initiatives such as Mom Connect. Most midwives (83.3%) admitted that using digital health helps them diagnose pre‐eclampsia accurately and detect early conditions such as pre‐eclampsia. In contrast to the findings, the qualitative findings revealed that the majority of women lack knowledge about the use of digital health in the early detection and treatment of pre‐eclampsia. The lack of knowledge among gravid women signals lack of support and poor implementation of available digital health initiatives by midwives in clinical practice. Utilisation of digital health has potential to transform the healthcare system and efforts must be made to utilise digital health to its full potential. Olu et al. (2019) affirmed with the results of the current study, that although digital health has the potential to improve the quality of care; lack of knowledge and awareness is a common challenge in using digital health in the healthcare system. The integrated findings indicated that although the midwives are knowledgeable about the utilisation of digital health, there is a gap in knowledge transmission and implementation of digital health initiatives through health education. Poor health literacy is associated with diverse negative outcomes such as less use of importance tools necessary for self‐care including digital health (Nawabi et al. 2021). Midwives should adopt a flexible approach to accommodate patients' knowledge (Downer et al. 2020). However, Ifeoluwa (2016) suggested that also the lack of knowledge about use of digital health could be associated with lack of skilled personnel. Therefore, there is a significant need of programmes to improve the knowledge of digital health among midwives and gravid women. Midwives should increasingly work towards collaboration between themselves and gravid women. Prioritise the needs of gravid women in utilisation of digital health (Park et al. 2021; Busse et al. 2022).

#### 3.3.2 Theme 2: Barriers Related to Digital Health Utilisation

To contribute to optimal quality care and equal health in the South African context, the challenges that hinder the implementation of digital health must be addressed. Below are the integrated barriers to the use of digital health in the early detection and treatment of pre‐eclampsia.

##### 3.3.2.1 Theme 2.1: Shortage of Digital and Human Resources

The shortage of resources remains a global challenge in the healthcare industry. The lack of human resources and materials hinders the provision of quality midwifery care (Mothiba et al. 2019). The quantitative study revealed that midwives face some impediments in providing quality care through the use of digital health. Impediments include things such as the shortage of human and digital resources. 72.5% of midwives revealed that the shortage of staff is a major problem in practice. Therefore, this could lead to too much workload. This was supported by 78.4% of midwives who noted that they have too much workload. Midwives (28.4%) reported that they barely cope with their workload and 21.6% reported that they barely manage to complete their work at the end of the day. 73.5% of midwives noted that there is usually a large number of patients in the facilities. However, this was supported by the qualitative findings of the study, which reported a lack of support and poor implementation of the available digital health initiatives by midwives. The majority of gravid women in the qualitative study reported that they did not receive support from midwives regarding the use and implementation of digital health. This could be because the shortage of human resources and too much workload has a direct impact on the quality of care, hence the lack of support of gravid women and poor implementation of the available digital health initiatives in the facilities. Furthermore, the integrated findings indicated that the shortage of human resources and digital resources is a common barrier to the use of digital health among midwives and gravid women. The majority of midwives (61.8%) in the quantitative study reported that their facilities do not have electronic devices such as tablets, Wi‐Fi devices or computers. This could be hindering the implementation of other available digital health interventions that may require the excess to the internet. Whereas the qualitative study reported that poor socio‐economic status impacts access to smartphones and network connectivity. Most of the gravid women reported not having smartphones, that data are expensive, and that they do not have access to Wi‐Fi. This was supported by a study conducted by Watkins et al. (2018) on the use of mobile health in rural areas, in South Africa. The study reported that most healthcare providers used their own phones for healthcare and paid the costs themselves, plus some healthcare providers developed informal digital health solutions in response to their work needs and lack of resources. Furthermore, the study reported that most patients reported a lack of financial ability to use digital health solutions.

##### 3.3.2.2 Theme 2.2: Power Supply Inconveniences Impacting Network Connectivity

The quantitative study pointed out that 72.5% of midwives experience electricity problems. This was supported by the qualitative study in which the gravid women reported that they experience a lot of load shedding and directly impact their network connectivity. Midwives (62.7%) reported poor network connections that could be the impact of the network connectivity problems of the power supply that subsequently resulted in poor access to digital health initiatives by midwives and gravid women. The study of Ifeoluwa (2016) corroborated that unavailability of constant electricity is a major challenge that does not only affect the healthcare sector only but also the economy. Similarly, a study conducted in Nigeria by Ifeoluwa (2016) on challenges in implementing digital health reported that the unavailability of power supply affects efficiency and internet accessibility. This concurred with Vodacom spokesperson and MTN SA's executive of corporate affairs said that if the power outage and back‐up batteries power at the cell towers is fully depleted, problems arise batteries begin to overheat and take longer to cool down, consequently affecting the efficacy of the network connectivity (Jacobs, 2021). However, this has a major impact on the implementation of digital health because during power outage, there is a possibility that the digital health might not function efficiently, and in some cases, infrastructures such as computers need power to function; during power outages, the digital health become inaccessible. Currently, South Africa is facing major electricity issues, currently characterised by consistent power outages or load shedding. This shows a need to create an infrastructure to make electricity and internet accessible to midwives and gravid women for them to implement digital health (Ifeoluwa 2016).

##### 3.3.2.3 Theme 2.3: Lack of Awareness of the Use of Digital Health in Early Detection and Treatment of Pre‐Eclampsia

The qualitative findings shown that gravid women lack awareness in the use of digital health, and most shown that they do not understand the use of digital health initiatives. They had to rely on alternative sources of information such as family and friends, brochures and magazines. Some of the gravid women revealed that their knowledge and awareness of utilisation of digital health, was only limited to the use of the internet and mom connect. Lack of awareness seems to be the impending factor for the use of digital health. This was supported by the quantitative findings, where 74.5% reported a lack of awareness of digital health utilisation. Ahmed et al. (2020) affirmed that lack of awareness, discomfort and lack of understanding and skills were the core reasons for not using digital health. The integrated findings confirmed that there is a lack of awareness of the use of digital health in the early detection and treatment of pre‐eclampsia. Although, midwives have knowledge about digital health, that does not necessarily mean that they are aware of the digital health initiatives or know how to use them. Hence, the lack of implementation of digital health by the midwives. The lack of awareness among midwives could be associated with the lack of policies, programmes and strategies to optimise the utilisation in digital health among pregnant women within the facilities. A study conducted by Ifeoluwa (2016) concurred with the results of the study that there is a lack of understanding of digital health as well as awareness, which affects the perceptions of digital health. Ifeoluwa (2016) further reported that the lack of awareness about the effective use of digital health could be the result of the government unwillingness to prioritise the use of digital health in rural areas. This is supported by the lack of sufficient policies governing the use of digital health in rural areas. This evidently show that the communities of low and middle incomes should familiarise themselves with digital health, so they learn the role and impact digital health in improving health. The role of awareness and education in promoting the healthcare workforce should be considered vital (Hoque et al. 2014).

##### 3.3.2.4 Theme 2.4: Lack of Training About the Use of Digital Health

The quantitative findings reported that although 81.4% of midwives received training on Pre‐Eclampsia, 94.1% of midwives received no training in the use of digital health within the facilities. Qualitative findings revealed that there is a lack of support and poor implementation of the available digital health initiatives by midwives. This could be possible due to a lack of training among midwives, which leads to poor implementation of digital health initiatives. In this study, the lack of training among midwives subsequently leads to incompetence in digital health utilisation consequently affecting the care received by gravid women. Utukuri et al. (2022) affirmed that inequities in digital literacy among the HCPs have to be noted, impending the use of digital health in clinical practice; this shows a lack of appropriate training. Curioso‐Vilchez and Coronel‐Chucos (2022) concur with Utukuri et al. (2022) that the lack of trained, qualified and skilled professionals is the main barrier to digital health utilisation, especially in low‐ and middle‐income countries. The integrated findings show that there is a significant need of the development of training programmes to training midwives on the use of digital health. This was supported by a study conducted by Utukuri et al. (2022), to not harm, HCPs must be sufficiently trained on the use of digital health in practice. Manyazewal et al. (2021) concurred with Curioso‐Vilchez and Coronel‐Chucos (2022) and Utukuri et al. (2022) that the adoption and implementation of digital health require extensive training of the HCPs; moreover, digital health implementation can further tackle the major clinical and public sector backlog further strengthening the healthcare ecosystem. The adoption and implementation to its full potential midwives should be trained in digital health (Manyazewal et al. 2021). Therefore, this clearly shows the need of training of the healthcare professionals in utilisation of digital health.

##### 3.3.2.5 Theme 2.5: Lack of Digital Health Initiatives Validation and Policies

Lack of technology validation hinders the use of digital health. The quantitative study revealed that 74.5% of midwives noted that lack of technology validation is a barrier to the use of digital health utilisation among midwives. This was supported by qualitative findings which revealed that the lack of validation of digital health initiatives is a concern. Gravid women reported a lack of trust and confusion in the use of digital interventions such as the Internet. Furthermore, the quantitative study found that there is a lack of policies, programmes and strategies to optimise utilisation in digital health among pregnant women within facilities.

Lack of regulation and policies impact the whole healthcare system. The healthcare is a sensitive field involving human lives. If digital health systems are implemented, rules and proper procedures should be devised and followed to ensure the safety of the individuals (Olu et al. 2019). Midwives are open to implementation of digital health initiatives as long they are trustworthy and scientifically study. Hence, the midwives stress the need for guidelines of utilisation of the digital health initiatives. The study of Fusheini and Eyles (2016) further substantiated that digital health can contribute in sustainable fulfilment of the UHC goals, only if there is digital health governance framework and it is implemented within a broader framework of the healthcare system. Curioso‐Vilchez and Coronel‐Chucos (2022) alluded that the lack of policies and strategies as well as evidence for scientific validity of digital health interventions is one of the main challenges to the use of digital health. Even though the national department of health issued a National Digital Health Strategy for SA (NDHSSA) 2019–2024. No Standard Operating Protocol or policies were designed and developed to govern the practice of digital health in the management of high‐risk pregnancies, such as pre‐eclampsia. The integrated findings revealed that there is a need of a regulatory framework and legislative to regulate the practice of digital health in clinical practice to enhance validation. This was corroborated by Cabuka (South African Medical Research Council, 2019) in the seminar on prioritising research and evaluation for digital health in SA; to support the development of digital health interventions there a need for formulation of a national legislative and regulatory framework for digital health. Furthermore, Cabuka (South African Medical Research Council, 2019) further affirmed that there is a required need to develop an enhanced digital health capacity and skilled workforce for support and implementation of digital health.

### 3.4 Integration of Findings With McKinsey 7s Model

The following paragraphs of the paper focus on the integration of the identified gaps and the McKinsey 7s model to develop evidence‐based strategies to enhance the use of digital health in the early detection and treatment of pre‐eclampsia by gravid women. However, only the components of the McKinsey 7s model that aligned with identified gaps will be discussed.

#### 3.4.1 Systems

The integrated findings of the study identified shortage of digital resources and human resources. The shortage of digital resources had an impact on the utilisation of digital health. The integrated findings revealed that there is poor infrastructure in the facilities. This includes the unavailability of tablets, computers, smartphones and Wi‐Fi among midwives and gravid women. Furthermore, the integrated findings indicated that power supply inconveniences impact network connectivity. The shortage of resources and power supply inconveniences disrupts the provision of quality care. The McKinsey model clearly state that the components are interconnected; however, the results revealed shortage of resources and power supply inconveniences which affects the system component of the model. This clearly shows that the balance within the facilities for the provision of quality care is imbalanced. Therefore, a change is required to address the shortage of digital resources and the inconveniences of the power supply to restore effectiveness and balance. Strategies 2 and 3 attempts to restore balance in the facilities.

#### 3.4.2 Staff

The integrated findings of the study indicated that midwives were not trained in using digital health. This could have been due to lack of support and poor implementation of digital health. Furthermore, in addition to lack of training, the findings revealed that a shortage of human resources (i.e., shortage of staff) affects the utilisation of digital health. Without adequate staffing, the implementation of digital health is affected. This may be affected due to factors such as poor midwife–patient ratio and overcrowding in facilities. Therefore, productivity and effectiveness in the provision of quality care is consequently affected. As a result, the staff component in McKinsey model is affected, requiring to make changes. Strategy 2 and 5 attempts to resolve the shortage of human resources and lack of training in the utilisation of digital health, respectively.

#### 3.4.3 Skills

The findings revealed a lack of awareness of digital health and inconsistencies in knowledge and the importance of this use of digital health. This clearly indicates incoherence among the components of the model due to lack of skills among the midwives possibly related to lack of training. Furthermore, it affects the competence of midwives in the use of digital health. Therefore, to restore balance according to the McKinsey model, the skills of midwives in utilisation of digital health should be improved. Strategy 1, 4 and 5 attempts to restore balance in skills of the midwives focusing on knowledge, awareness and training, respectively.

#### 3.4.4 Style

The attitude of senior management establishes the code of conduct. In the context of the study, it is the responsibility of the higher management and policymakers to ensure that validated digital health initiatives are made available to the employees and policies supporting such are also provided to the employees. However, the integrated findings identified that lack of validation of digital health initiatives impacts the use of digital health in clinical practice. This is said to be related to the lack of policies and protocols for the use of digital health. Therefore, the attitude and practices of higher management and policymakers consequently affect the duties of midwives in using digital health. The style component of the McKinsey model is affected, resulting in ineffectiveness in the facilities. Strategy 6 attempts to restore balance by addressing the styles of leaders towards use of digital health.

### 3.5 Evidence‐Based Strategies to Enhance the Utilisation of Digital Health in Early Detection and Treatment of Pre‐Eclampsia

The summarised strategies are shown in Table 5 below along with the performance drivers.

**TABLE 5:** Strategies to enhance digital health utilisation in early detection and treatment of pre‐eclampsia (Ngwenya, 2023).

Strategy	Responsibility and performance drivers	
	Midwives	Stakeholders and policymakers
Prioritisation of maternal digital health interventions aimed at improving the knowledge of midwives and gravid women on the utilisation of digital health to early detection and treatment of pre‐eclampsia	Prioritise the needs of gravid women by updating themselves with knowledge and the importance of digital in the early detection and treatment of pre‐eclampsia to transmit that knowledge to gravid women Facilitate the knowledge of gravid women about utilisation of digital health to ensure the safety and accurateness of information used by gravid women from digital health platform Attend workshops, seminars and training to keep themselves updated about digital health Provide informational support about digital health to gravid women Involve the designated birth companions when teaching gravid women about digital health; upon women's request Implement the available digital health initiatives to enhance the knowledge of gravid women	The political obligation that places digital health at the centre of maternal health to achieve sustainable development goal 3 and universal health coverage goals Keep midwives informed on current knowledge with regard to digital health Establish communication channels that can be used by midwives to enquire about digital health interventions Reinforce the vision and mission statement of implementation of digital health in clinical practice
2 Provision of sufficient human and digital resources to ensure provision of optimal quality care to pre‐eclampsia gravid women	Keep inventory and ensure maintenance of the digital resources provided Use the provided digital resources for their intended use The operational manager should organise in‐service training to ensure that the midwives are trained on the use of the provided digital resources Adhere to departmental policies and guidelines in purchasing and repairing of the digital resources	Provide midwives with necessary digital resources require to fully implement digital health in clinical practice Employ more trained midwifery practitioner to provide optimal midwifery care through digital health Follow‐up on provided digital resources on their adequate functioning Provide 24/7 wireless Wi‐Fi access to digital health initiatives with restricted access to non‐related digital websites
3 Development of a comprehensive plan aimed to address network connection issues following power outages	Educate and guide the gravid women on digital health apps that don't require a network connection to use	Develop digital health initiatives that can be accessed offline Purchase high‐power batteries that can sustain the cell towers for more than 24 h Develop and implement more messages‐orientated mobile health initiatives
4 Improvement of the awareness measures on utilisation of digital health to optimise quality care among pregnant women having pre‐eclampsia	Midwives must uphold ethical values and respect of the gravid women to establish a therapeutic trusting relationship between the gravid women and midwives to promote openness and trust Ensure and create informational support programmes for gravid women at the primary level to guide and inform gravid women on utilisation of digital health in early detection and treatment of pre‐eclampsia Educational support of families and communities to further promote the utilisation of digital health in the early detection and treatment of pre‐eclampsia Actively involvement and collaboration with communities through awareness campaigns to educate on the benefits of digital health in pregnancy	Monitor and evaluate the practices of midwives on the implementation of digital health Innovate and develop patient‐centred educational guidelines on the use of digital health by gravid women Develop and implement national awareness strategies on use digital health. This could be done through broadcasting in TVs and radios
5 Development of context‐rich training programmes to advance the skills and competency of midwives on the utilisation of digital health	Demonstrate commitment in advancing skills and knowledge in nursing informatics Attend seminars and workshops to advance their skills and knowledge about digital health	Develop and Implement nursing informatics graduate programmes Provide and allocate a budget specific for the training of midwives in digital health Monitor and evaluate the competency of midwives in the implementation of digital health in practice following training
6 Provision of mandatory jurisdictive and regulatory framework stating the authorised digital health initiatives	Implement and practice within the provided policies and regulatory framework of digital health Implement the authorised digital health initiatives in clinical practice Provide feedback on the authorised digital health initiative to policymakers for review purposes	Adopt a mandatory jurisdictive and regulatory framework within which midwives can practice and teach gravid women about the importance of the utilisation of digital health in the detection and treatment of pre‐eclampsia Adopt national and provincial quality standards that give direction of utilisation of digital health within maternal health practice by healthcare professionals, further strengthen the maternal digital health systems Develop a comprehensive national digital health policy to govern digital health in practice Adopt and encourage access to authorised digital health environment for gravid women focusing on common complications following pregnancy Introduction of social media digital platform involving health professionals and gravid women focused in maternal health

## 4 Discussion

The main study was conducted with the primary purpose to develop strategies to enhance utilisation of digital health in early detection and treatment of pre‐eclampsia by gravid women using a mixed method. The mixed methodological inquiry identified six vital areas for developing the evidence‐based strategies to enhance the utilisation of digital health in early detection and treatment of pre‐eclampsia by gravid women. Nonetheless, the evidence‐based strategies focus on supporting the midwives and stakeholders/policymakers in optimising digital health utilisation in early detection and treatment of pre‐eclampsia by gravid women. The newly developed strategies are a step towards accessibility of optimal quality maternal healthcare services through digital health among gravid women diagnosed with pre‐eclampsia in terms of the Universal health coverage goals. As South Africa thrives moving towards the universal health coverage for better access to healthcare services through healthcare systems (Arnaert et al. 2019). In addition, DH initiatives such as mobile health technologies were developed to improve better access to communities in low‐ and middle‐income countries.

However, lack of knowledge, poor socio‐economic status and necessary human and digital resources openly disturbs the quality of maternal healthcare services offered further leading to catastrophic maternal and perinatal outcomes. Therefore in order to maintain high quality standard midwifery care, this paper suggest it is of consequential to facilitate proper primary interventional strategies and treatment modalities that can ensure quality care. This can be ensured through continuum educational development of the personnel rendering healthcare services from primary to tertiary level and through provision of necessary digital and human resources. For SA to achieve the SDGs 3 which is to ensure healthy lives and promote well‐being for all ages (Fusheini and Eyles 2016; Rambire 2022). It is solely dependent on the proper allocations of sufficient finance for resources, enhancement of knowledge of gravid women and educational development programmes for the midwives. Also through emphasis of professional growth daily through supervised in‐service training in each and every facility. The Sub‐Saharan African countries suffer a great deal loss of gravid women during perinatal, intrapartum and postpartum due to pregnancy complications such as pre‐eclampsia. Therefore, it is imperative to apply the developed strategies as they could possibly effect a great deal outcome on the maternal healthcare systems.

### 4.1 Limitations

The study only involved midwives and pregnant women. Therefore, the strategies were developed only from those participants. Moreover, the study was only conducted in primary healthcare facilities and they cannot be generalised to other context. Lastly, this strategies were not validated.

## 5 Conclusion

Although the study focused in the primary healthcare facilities in Mpumalanga province. The developed evidence‐based strategies promote the breaking of the status quo in the current practice in the public healthcare industry of South Africa. Transitioning from traditional clinical practice to digital health practice could possibly improve the quality of care received by gravid women and most likely improve the knowledge of the gravid women with regard to their conditions. Furthermore, their implementation could possibly improve the proper practicing and implementation of digital health in clinical practice.

## Author Contributions

M.W.N. participated in contextualising, designing and data collection of the study. L.M. and T.M.M. supervised the study. M.W.N. and L.M. participated in analysis and interpretation of the findings. M.W.N. drafted the manuscript. L.M. and T.M.M. reviewed, edited and finalised the manuscript. All authors read and agreed on the final manuscript.

## Ethics Statement

This study forms part of a larger study which was approved by the Turfloop research ethics committee in the University of Limpopo, South Africa (TREC/81/2022: PG). The permission was granted by the department of health Mpumalanga province with the support of the district manager.

## Consent

The ethical principles of social justice, beneficence and non‐maleficence, informed consent, confidentiality and anonymity were observed and adhered to throughout the study. A detailed informed consent was given to all participants to sign prior data collection and was clarified and described as such.

## Conflicts of Interest

The authors declare no conflicts of interest.

## Data Availability

The data sets used during this study are available on request for readers from corresponding authors.
